# Correction: Pinging the brain with visual impulses reveals electrically active, not activity-silent, working memories

**DOI:** 10.1371/journal.pbio.3001603

**Published:** 2022-03-25

**Authors:** Joao Barbosa, Diego Lozano-Soldevilla, Albert Compte

In Fig 3, the inset is uninterpretable and should be removed. The derivation of the shuffle predictor for this data is compromised due to baselining in the interval [-200 ms, 0], a period containing traces of the memory code of the unattended stimulus. This baselining introduces a spurious code aligned with the decoding of interest, so that random permutation of stimulus labels cannot properly estimate the relevant shuffle predictor.

The authors have provided a corrected version of Fig 3 here and amended the caption accordingly.

**Fig 3:**
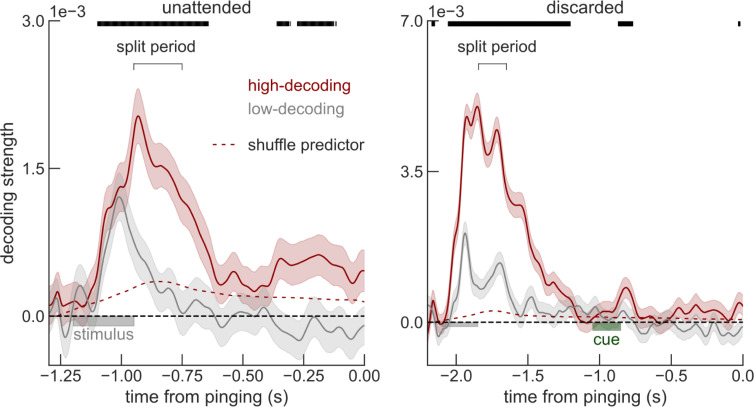
Sessions with high early-delay (split period, Methods) voltage decoding have a sustained code for unattended memories (left, red), but not for discarded memories (right). Error bars are sem. Decoding strengths from high-decoding sessions were compared to the shuffle predictor (top black bars mark significant deviation, one-sided p<0.05, Methods). Time course and data are similar to Fig 1A and 1B. Data from Wolff and colleagues (2017) [7].

In the Results subsection ‘Lack of statistical power suggests spurious evidence for silent representations of unattended memories,’ sentence 9 should be removed. The correct sentence series is:

We found that unattended memories could be robustly decoded during the whole delay (0.25–1.2 s, p = 0.002 randomization test, Methods) and in particular immediately before pinging (250 ms window, p = 0.039, randomization test, Methods) from high-decoding sessions, while discarded memories could not (both p>0.45, Fig 3). Note that we used one-sided statistical tests (Figs 2 and 3), since negative decoding strengths are not expected.
