# Supplementary figures and images for: Pinging the brain with visual impulses reveals electrically active, not activity-silent, working memories
Source: PLoS Biol. 2021 Oct 21;19(10):e3001436. doi: 10.1371/journal.pbio.3001436 (PMC8641864; doi:10.1371/journal.pbio.3001436)

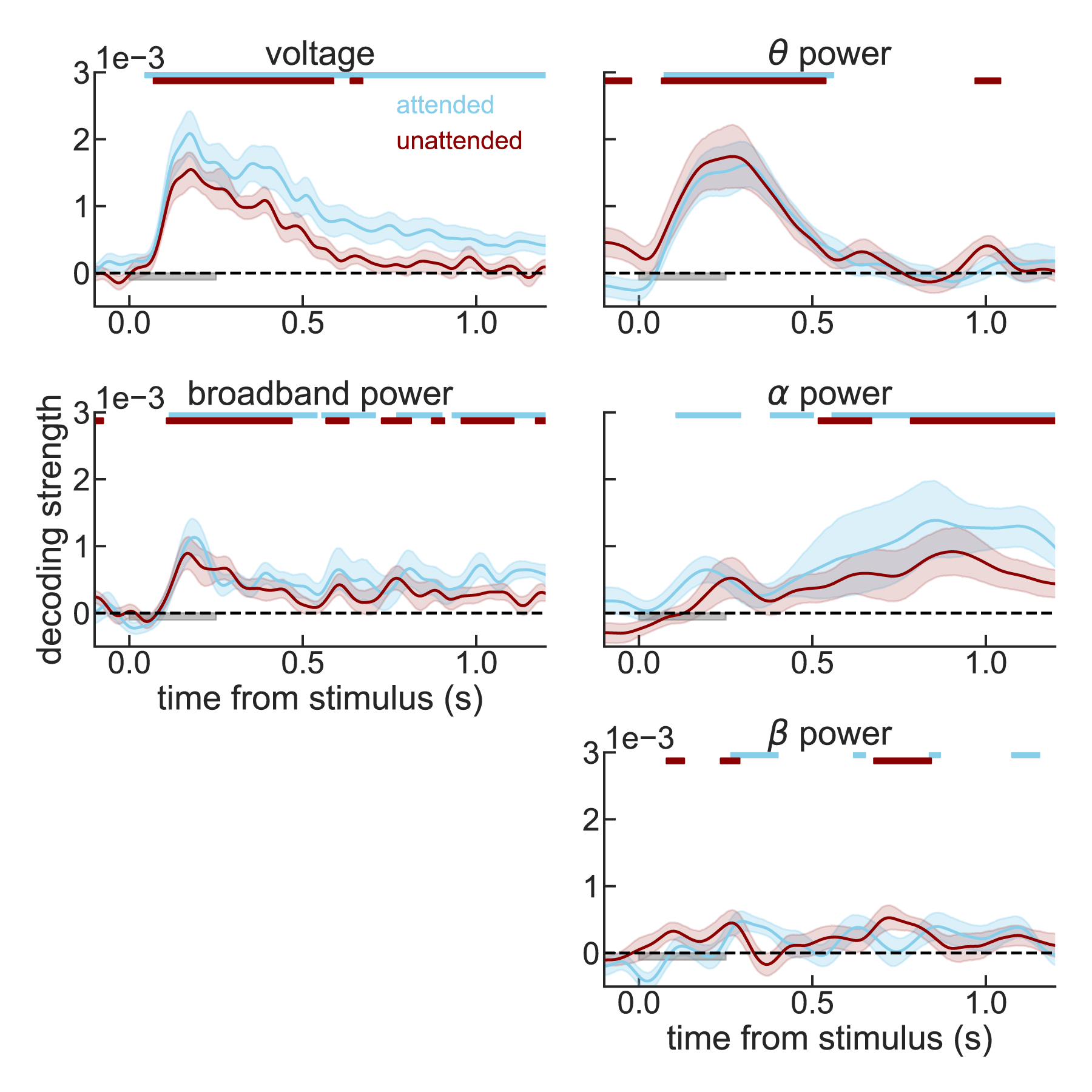

Supplement: S1 Fig — Data from Wolff and colleagues (2017) [7]. (TIF) [file pbio.3001436.s001.tif]

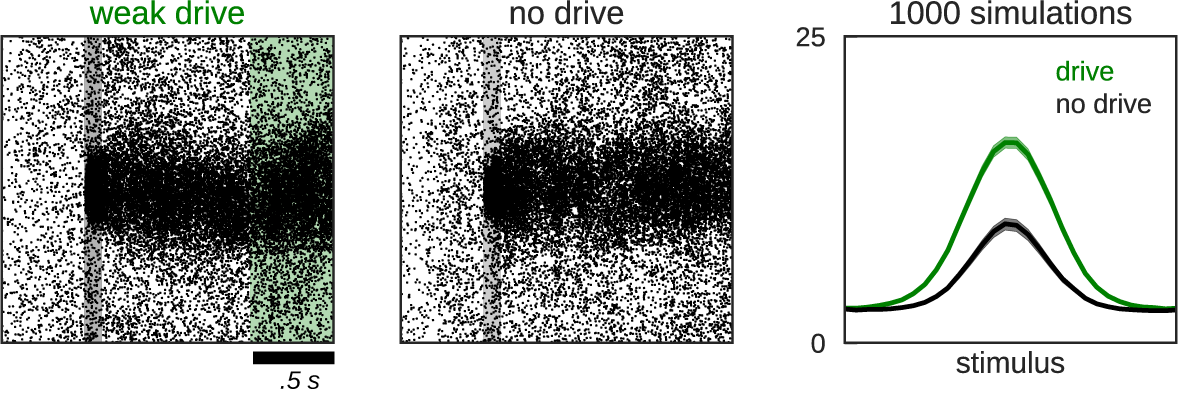

Supplement: S2 Fig — Two example stimulations of a bump-attractor with (weak drive) and without (no drive) a nonspecific drive at the end of the trial. Importantly, we did not include short-term plasticity in either simulation; thus, reactivations are not possible. Right, tuning during the last 0.5 s is higher for the trials in which a nonspecific drive was delivered (green), compared to when no drive was delivered (black). Similar models have been put forward in previous publications [29] (see also [39]). (TIF) [file pbio.3001436.s002.tif]

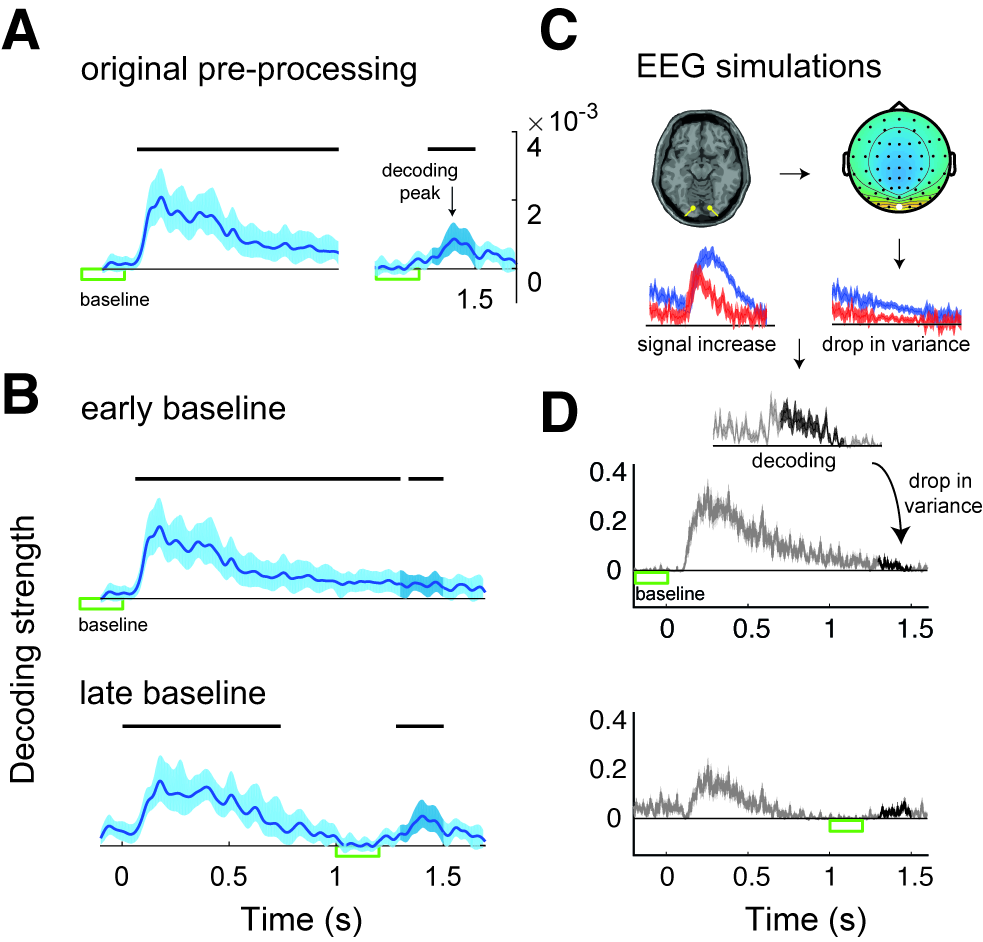

Supplement: S3 Fig — (A) Original data from Wolff and colleagues (2017) [7] was baselined twice. (B) For our analyses, we de-baselined the second baselining of each trial so we could get continuity in EEG voltage traces through the whole trial (early baseline). Importantly, the exact time of the baseline affects the strength of “reactivations.” Note that data with early baselining do not show any visible increase in EEG decoding. (C) Top: diagram outlining the computer simulation that generated the EEG synthetic data, as if 2 current dipoles were placed within the visual cortex (yellow) to recreate artificial trials. Two different sets of trials (n = 200, blue and red), corresponding to 2 different stimuli (e.g., 0°, 45°) were generated (Methods). We simulated 2 event-related potentials (electrode “Oz”; white) followed by a drop in across-trial variability. Bottom: illustration of how a similar signal-to-noise ratio increase (shown in “decoding” for the drop in variance case) can result from a drop in variance or actual reactivation (signal increase). (D) Through simulations, we show that the baselining procedure introduces spurious reactivations in data without any true reactivation signal. By applying the decoding methods of Wolff and colleagues [7], we observe that (as in the data) spurious reactivations are barely visible with a distant baseline (early baseline) but are amplified for more proximal baselines (late baseline). These analyses illustrate that baselining is problematic and should be avoided, as it has been previously pointed out [28]. Darker lines mark the impulse period in the data and drop in variance in the simulations. Data from Wolff and colleagues (2017) [7]. (TIF) [file pbio.3001436.s003.tif]
