# Supplementary material for: Pinging the brain with visual impulses reveals electrically active, not activity-silent, working memories
Source: PLoS Biol. 2021 Oct 21;19(10):e3001436. doi: 10.1371/journal.pbio.3001436 (PMC8641864; doi:10.1371/journal.pbio.3001436)
Supplement: Attachment — Submitted filename: Barbosa_et_al_rebuttal.pdf [file pbio.3001436.s004.pdf]

# Point-to-point reply to the Reviewers’ criticisms

We thank the reviewers for their constructive criticisms, which have led to a thorough revision of this manuscript, with significant improvements. The manuscript now makes a crisper point about the fundamental finding, the decodability of unattended memories during the delay period, and it adds a deeper understanding of the discrepancy with previous interpretations. Substantial reanalyses of the data now attribute this discrepancy to limited statistical power in the dataset (when analyzing EEG voltage decoding), as opposed to a true qualitative difference on the signals revealed by alpha and voltage decoding. With this among other changes, this revised manuscript now addresses all the criticisms of the Reviewers, which we detail point-by-point in the following (our text in blue):

**Reviewer #1, Brad Postle:** "Unattended short-term memories are maintained in active neural representations," Barbosa, Lozano-Soldevilla, and Compte. This manuscript present the reanalysis of data from two publications (from other groups) about which, as the authors write, "The[ir] interpretation of reactivations from absent decoding as evidence for 'activity-silent' storage has had a strong impact in the field." The major findings are threefold. First (1), reanalysis of the data from Wolfe et al. (2017) replicated the original finding of failing to decode evidence for an active representation of the unprioritized memory item (UMI) when the EEG data were analyzed in their voltage format, but SUCCEEDED in decoding evidence for an active representation of the UMI from the alpha-band component  of the signal, after these same data had been spectrally transformed. Second (2), they show with simulations, then empirically, that the effect of the visual "ping" is to decrease trial-to-trial variability in the signal. Third (3), they show with simulations, then with empirical analysis of the data from Rose et al., (2016), that the effect of a pulse of TMS is the opposite of that of a ping: TMS increases trial-to-trial variability. The significance of (2) and (3) is that (2) may explain why the effect of the ping on time-voltage data is to briefly rescue decoding of the UMI, whereas (3) would seem to indicate that the activity-silent interpretation of TMS-evoked rescue of decoding of the UMI remains viable (although, as this review will highlight, this interpretation is equivocal in the manuscript as currently written). This manuscript will be of considerable interest to many who study working memory from a variety of perspectives (computation, behavior, intra- and extracranial electrophysiology, fMRI, neurostimulation…), because of its important implications at both empirical and theoretical levels. (As the senior author of Rose et al. (2016) I can't not forgo anonymity and still write a comprehensive review.)

This work is important and will get a lot of attention, but I worry that some of the framing fails to emphasize some of the most important elements and pushes a narrative that's both too strong and somewhat out of date. Let's start with the title - it's stating proposition that has already been described on several occasions

We thank the reviewer (Brad Postle) for this observation. We realize that the title was indeed not properly portraying the content and scope of the reported findings. Following the reviewer’s suggestion, we have now changed the title to reflect more accurately the precise content of the study: *“Visual pinging reveals active, not silent, working memories”*

, including in one paper that they cite, but not in this context (Christophel et al. 2018),

Based on this comment of the reviewer, and also on observations of reviewer 3, we have realized that we were not fully crediting the relation of the paper by Christophel et al. (2018) with our new

results. We are now extensively citing this work and we have included two new figures (Figs. 2,3) inspired by this paper (and the comments of reviewer 3).

and several that they don't (van Loon et al. 2018, Wan et al. 2020, Yu et al. 2020, Libby and Buschman 2021). This latter group all document a phenomenon that the decoding methods used here can't distinguish, but that the authors might consider investigating with a different method, which is that the active representation of the UMI is transformed from its format when it is a prioritized (P)MI. (Two of these papers are from my group, and I want to be clear that I have no expectations that the authors need to cite them.)

We completely agree with the reviewer that these works are relevant for our manuscript, as they already demonstrate that unattended memories in various paradigms are encoded in electrically active activity, decodable from EEG or directly with microelectrodes. Thus, our analyses in this manuscript reconcile the influential study of Wolff et al. (2017) with more recent, convergent evidence. We make this point now in the Discussion:

> "Our data reconciles the influential study by Wolff et al. [7] with recent works showing unattended memories actively encoded in scalp EEG [32–34], in the activity of cortical association areas using large-sample fMRI analyses [30] or intracranial recordings in monkeys [22,49], and in neural activity in visual areas of rodents [37]. "

Also, as per the reviewer's suggestion, we have looked into the format of unattended (UMI) and attended (PMI) memories and we do not find evidence of inverted coding. To investigate this, we computed "tuning curves" by averaging alpha power across stimuli (analyses 1). In an alternative analysis (analyses 2), we also computed the std across stimuli for each electrode as a rough measure of code strength for each electrode. We then correlated both these vectors of PMI vs UMI for each subject (n=500). For both analyses, we find a positive correlation between the representations (r>0.3) but only one session stronger than correlating codes computed from shuffled stimuli (p=0.01, p=0.03 t-test against shuffle). This means that electrodes tend to use the same code for PMI and UMI (analyses 1) and that similar electrodes encode PMI and UMI (analyses 2). Both cases do not support an inverted code (for session 1, inconclusive for session 2), which would predict a negative correlation. Because this is not the focus of our paper we opted to not include this analysis as it would imply discussing a topic that was not addressed in the current manuscript.

Furthermore there's an implication here and in many parts of the manuscript that the UMI in the Wolfe et al. studies is NOT also stored in an activity-silent format, but of course the authors can't know that.

We fully agree with the reviewer that we do not have any evidence that long-lasting cellular or synaptic mechanisms are not participating in active network storage. Also, we do not think this is possible and there is evidence for the contrary (Barbosa et al. 2020). We also agree that we were not making this point clear enough in our previous manuscript, as the reviewer rightly points out. We have therefore included explicit references to this, in particular (1) at the end of the introduction ("Finally, we argue that the increase in stimulus decodability following an unspecific stimulus, seen in human [6,7,18–20] and monkey electrophysiological experiments [21,22], can be explained by network models with or without short-term plasticity based on ongoing active, not silent, neural representations").

(Finally, with regard to the title, the authors of both (Wolff et al. 2017) and (Rose et al. 2016) refer to their tasks as "working memory" tasks, and the authors might consider using this label, which is much more widely used in the field.)

We thank the reviewer for this remark. This issue is now solved with the new title.

This false dichotomy (active vs. activity-silent) is particularly dissonant in the final paragraph, in which the authors seem to ignore their own (very elegant) work that showed compelling positive evidence for activity-silent representations in the PFC of the monkey. Also important to consider are different results from the monkey PFC in which a "cognitive ping" reveals otherwise undetectable (and so 'probably' activity-silent) representations of stimuli (Stokes et al. 2013).

We thank the reviewer for pointing this out, we have now stated explicitly our view in the manuscript in particular in relationship with our previous work in the concluding sentences:

> “we [5] and others [56] have found ...”
>
> “We have recently reported that [5], between consecutive trials — when the previous memory should be discarded, similarly to uncued memories in —, neurons fire more synchronously after having been engaged in active working memory storage, suggesting that discarded memories can leave involuntary silent traces. ”

In regard to Stokes et al. 2013 we want to clarify that the mentioned “cognitive pinging” is not after a truly undetectable representation. By comparing the baseline decoding prior to trial start (t<0 in their Fig. 2), where there is really no information, to the baseline decoding before the cognitive pinging condition (t~0 in their Fig. 5), one notices immediately that these are two different situations. Indeed, the memory appears to be decodable before the pinging, albeit at a low baseline level. This is an important detail, as external stimulus can increase decodability even in networks without short-term plasticity, thus unable to do proper reactivations. We have now included new simulations that show this in one new figure (S3; see also Fig 4C), and we cite this work in relation to that model.

Finally, as the authors (seemingly grudgingly) acknowledge, the most straightforward conclusion from their reanalysis of the data from Rose et al. (2016) is the UMI in that study most likely was held via activity-silent mechanism.

In the first paragraph of Discussion, we now acknowledge that in view of our study, activity-silent storage is the most straightforward explanation for TMS:

> “Based on the difference in behavioral impact of these two perturbation protocols (visual pinging does not affect working memory behavior, but TMS does), we speculate that visual pinging may increase EEG decodability via reduced across-trial variability or by transient boosting of active attractors, while TMS-induced reactivations would be supported by activity-silent mechanisms. Note that temporarily boosting an active attractor should not have a strong impact on behavior beyond the boosting period (unless additional long-lasting cellular mechanisms are engaged), while true reactivations from activity-silent stores should have a long lasting impact, as the silent trace is refreshed.”

However, based on the specific comments of Reviewer #3 regarding the difficulty of interpreting trial-by-trial variability changes in TMS studies (possibly trial-by-trial variability in the location of the coil), the support of our data for the activity-silent hypothesis of TMS reactivations should be treated with caution. Following this combined advice, we are now explicitly stating in Results how we conclude in relation to the neural basis of TMS reactivations, but adding a note of caution:

> “Such increase in across-trial variability is in accordance with the activity-silent working memory model presented before (Fig. 4C, black), thus supporting the interpretation of TMS EEG reactivations as signals recovered from activity-silent traces [5,6]. However, a note of caution is in order: the difficulty in precisely locating the TMS coil in different trials may strongly contribute to increased EEG variability, which could mask the true effect of TMS on EEG variability.”

Finally, it need not be the case that the idea of activity-silent representation "stands in contradiction with computational models of 'activity-silent' storage, where short-term plasticity changes are induced by neuronal activity" if one allows for existence of cognitive control. Indeed, Jacqueline Fulvio in my group has show behaviorally that the 'behavioral reactivation' TMS effect is subject to control (Fulvio and Postle 2020). (Again, I'm not asking for citations, but it is the case that I work in this area …) Masse et al. (Masse et al. 2019) and Manohar et al. (Manohar et al. 2019) have demonstrated that the two formats can, in principle, co-exist, and if the authors embraced this idea they could preempt readers like me getting distracted by an issue that's not the main point of the results.

We realize that our wording of that sentence was unfortunate and could be read to mean that “activity-silent” mechanisms are in contradiction with activity-based memories. We reformulated that sentence so it is now clearer in its meaning: “The mechanisms for such selective switching of activity-silent memory are unclear, as in existing computational models of activity-silent storage [1,2,4,5,16,17] short-term plasticity changes are induced by neuronal activity, regardless of its behavioral relevance”. With this we mean that there is no current biophysical understanding of how short-term plasticity may be loaded or not loaded based on behavioral relevance (maybe neuromodulation could do it, but it is not at all obvious). More work is needed to propose some plausible mechanism for this hypothesis. The second paragraph of Discussion discusses the interaction between activity-silent mechanisms and ongoing activity (“While there is extensive evidence for long-lasting cellular and synaptic mechanisms...”), and it explicitly endorses the idea that these mechanisms co-exist and cooperate in the brain.

I realize that I spilled a lot of ink to make this point, but it is really the only big-ish concern that I have with this otherwise excellent paper. I'll make more specific comments in the order in which they appear.

2nd paragraph: Rose el al. (2016) also reported an inability to decode the UMI from spectrally transformed data (Figure S5, which also show that the PMI is decodable from alpha and the UMI reactivation from beta).

We thank the reviewer for drawing attention to this piece of evidence. We have difficulty interpreting this figure based on the ambiguous timing (in relation to the TMS pulse time) of the UMI reactivation

in beta. Also there could be some indications that low statistical power may also affect some of the statistically non-significant points that appear to hover over the chance line prior to the TMS pulse. As our manuscript now emphasizes low statistical power (rather than voltage versus alpha) as a fundamental consideration when assessing decodability to validate activity-silent memories, our sense is that this should also affect how we value the evidence from this figure. This caution must be also framed within the recent reports of active representations for unattended memory items (Christophel et al. 2018, van Loon et al. 2018, Wan et al. 2020, Yu et al. 2020, Libby and Buschman 2021, Panicchello and Buschman 2021). Furthermore, the other reviewers explicitly asked to tone down our conclusions in relation to TMS reactivations. Taken together, all this underscores our cautionary take now on the interpretation of TMS-induced EEG reactivations. While an activity-silent working memory substrate is still possible based on the variability analysis that we provide, there is the possibility to explain that based on TMS coil location variability across trials.

Results and Discussion: The first time the key result is mentioned "We found that a sustained alpha power code tracks the items that remain relevant for future behavior" it'd be helpful to specify that it's tracking orientation; during my first read-through I was uncertain whether it was orientation or location-on-the-screen that was being decoded.

Thanks for this very pertinent observation. We have now clarified early on that we are decoding orientation, not location (2nd sentence in Results: “Thus, we analyzed alpha-power information content for attended, unattended and no-longer relevant orientation memories in the publicly available dataset of the original publication by Wolff et al. [7].”)

"Furthermore, it challenges the current view on the role of alpha power during working memory maintenance [18], which would suppress immediately irrelevant memories [19]." This is unclear, because 18 argues against a suppression/inhibition for alpha?

We agree that this sentence was too cryptic and would have required a more extended discussion section on the role of alpha signals in working memory, for which there is extensive literature. In the current manuscript, this debate is now not pertinent, since we found that decoding of unattended items is also significant in the raw voltages, so a specific role for alpha as opposed to EEG voltage is not really a topic to discuss. Our data supports an underlying active neural substrate for unattended items, picked up both by EEG voltage and alpha. There is nothing specific about alpha in our analysis, it just appears to be a signal more robust to EEG drifts for our decoding purposes. Therefore, we have removed this sentence from the manuscript.

This clause needs more unpacking "possibly reflecting the prioritization of strong competition between actively held memories in attractor networks [20,21]" because someone unfamiliar with the details of 20, 21 won't necessarily understand what is meant here.

We thank the reviewer for noticing this rather cryptic sentence and we have now fully unpacked it in the text (“These neural dynamics could result from competitive interactions between prioritized and unprioritized memory items, in line with competing attractors in networks without activity-silent mechanisms [20,24,26].”)

Is the work of Bae and Luck (recovery of decoding of previous trial's stimulus during the next trial) also relevant here, perhaps as part of a more detailed consideration of the implications of (Barbosa et al. 2020)?

We have considered the reviewer's suggestion. We now cite this manuscript (Bae and Luck, 2019) as an example of EEG reactivations and how it relates to our work:

> "To avoid confounds with active representations, we [5] and others [56] have found evidence for activity-silent traces from previously memorized but now irrelevant items, for which chance-decoding is expected in principle..." and the rest of the paragraph

Additionally, we now discuss the work by Bae and Luck (2018) related to decoding from alpha versus raw voltages, which we think is more directly related to our results.

Data preprocessing: it's unclear what is meant by the words "to revert this baselining." Additionally, a sentence or two explaining in more detail how this was done, and why it was important to do, would be helpful.

Following the remarks by several reviewers, we have now included an extra figure (Sup Figure 3) to explain clearly what baseline we reverted and what are the consequences of using it.

"funcion" is not English.

Corrected. Thank you.

Figure 1: this is picky, but "decoding from alpha power (Methods), which reveals a strong sustained code of the unattended stimulus" strikes me as imprecise. What's being revealed is a stimulus representation, and from that one infers that there is a code that supports this representation, right?

We rephrased the aforementioned sentence to "decoding from alpha power (Methods), which reveals a sustained representation of the unattended stimulus." We thank the reviewer for this remark.

Figure 2: "A signal-to-noise ratio increase can reflect a drop in variance …" Isn't it meant that an SNR increase can result from a drop in variance?

We thank the reviewer for this remark. We rephrased the sentence using the reviewer suggestion.

Signed, Brad Postle

Thank you Brad for your in-depth reading of our manuscript and for the very insightful comments. They have prompted significant improvements in the revised manuscript. We have included your name in the Acknowledgments section. Please let us know if you would rather not be named there.

**Reviewer #2: Summary:** The authors investigate whether they can decode information that was previously thought to be maintained in silent working memory. The authors ask this question by using alpha power at posterior electrodes to decode working memory representations. In at least one experiment, they are able to decode working memory information. Furthermore, the authors then suggest that visual "pinging" may change the signal-to-noise ratio of EEG activity, thus increasing decoding accuracy of already active neural representations (by increasing SNR), rather than re-activating latent traces. Many have pointed out already that absence of evidence is weak evidence for absence of decodable memory activity, and this study is an important example of this point. Given the strong influence of the "activity silent" models of working memory storage, the

present findings provide a critical alternative explanation for one of the more prominent studies arguing in favor of activity-silent modes of storage in working memory. The present authors also report simulations that support their hypothesis regarding the SNR effects of visual "pinging" (i.e., presentation of an irrelevant visual stimuli), showing that pinging may reduce across trial variability and thus increase SNR. This adds strength to the authors' speculations that the putative "silent" representations may have simply been masked by noise rather than truly silent.

However, the authors' account doesn't provide a direct explanation for why it was the *relevant* and not the irrelevant memory representation that was decodable in the raw voltages after visual pinging. That is, while it is clear that they have successfully decoded using alpha power, why would visual pinging only resurrect the relevant item's representation if the authors are correct about the effects of pinging on across trial variability? Those variability effects should influence decoding of both relevant and irrelevant items, shouldn't they? The authors assert that "…alpha power tightly tracks working memory contents, *regardless* of their immediate behavioral relevance." (p. 3, results and discussion), but I thought figure 1 was ambiguous on this point. 1C shows a trend towards higher decoding strength for the relevant item, especially at the end of the delay period.

We agree with the reviewer that our results show a clear difference between irrelevant (or uncued) discarded memories and unattended but relevant memories. We thank the review for noting this unclear but important point and we want to make it clear that this result is in line with our message and does not reflect an inconsistency in our arguments. We have now included a new set of analyses in two new figures (Figures 2,3) and an accompanying paragraph to address this point that we hope will make the message clear starting in “In line with the hypothesis of an active but weaker representation of the unattended items, recent studies [27,28]“

Figure 1D also seems to show a divergence of the relevant and irrelevant decoding strengths at 1sec, but I wasn't sure why the irrelevant item line was dashed instead of solid (perhaps this is indicated somewhere in the manuscript, but I couldn't find it. I'd recommend making this more clear in the figure caption).

We thank the reviewer for noticing this missing piece of information. The dashed line is indeed marking periods in which the decoded items have become behaviorally irrelevant by an instruction cue (discarded memories). This condition occurs only for experiment 1 (Fig. 1B,D). We have added this information in the caption.

This then leaves the mystery of why only the relevant item appears to be tracked by EEG voltage following the ping. The authors do state, "However, while attended memories are decodable both in alpha power and voltage traces, unattended memories are only detected in alpha power, *possibly reflecting the prioritization of strong competition between actively held memories in attractor networks.*" Does this sentence imply that the traces reflected in EEG voltage were indeed "silent" prior to the pinging? I thought this was unclear from the manuscript.

We thank the reviewer for noticing this unclear message. Indeed, we do not mean that memories were stored in silent traces. We mean quite the opposite, they are both stored actively, but one of them (unattended) in a slightly weaker code. Because it is a weaker code, it is undetectable in more noisy measures (raw voltages) but detectable in more robust measures (alpha power). We are now emphasizing this idea at various points in the manuscript, supported in addition by new analyses (see point immediately below):

1st paragraph in Results:

"In this view, attended items would be represented by strong neural signals (represented both in voltage and in alpha power) while unattended items would be kept in analogous but weaker neural signals (picked only by alpha power). These neural dynamics could result from competitive interactions between prioritized and unprioritized memory items, in line with competing attractors in networks without activity-silent mechanisms [20,22,23]."

1st paragraph in Discussion:

"In addition, we verify that representations for unattended items are notably weaker than for attended items, consistent with biased competition between active memories [22]."

The later arguments about pinging reducing across trial variability seem to leave open the possibility that both attended and unattended items were represented in EEG voltage, but with SNR too low to confirm it with the original analysis pipeline. I think this points merits careful clarification in the manuscript.

We thank the reviewer for suggesting to make this point clearer. As per this Reviewer and Reviewer #3 suggestion, we have now performed new analyses to address the impact of statistical power on decoding analyses from raw EEG. In order to increase SNR in raw EEG analyses, we have (also in second paragraph of Results):

1) smoothed the voltage traces with a 32-ms kernel prior to decoding (instead of smoothing instantaneous decoding accuracies as done in the original study [7]; Methods), 2) averaged decoding accuracies over an interval of 200 ms before the pinging impulse, and 3) pooled trials from all sessions and subjects (Methods).

Although with these preprocessing steps decoding remained non-significant, we could establish a clear qualitative distinction in how the effect size depended on sample size for unattended memories compared to the likely absent neural representations of discarded memories (new Fig. 2).

We have also simulated an increase of signal-to-noise ratio by removing sessions with overall low decoding (included in a new figure (Fig 3)) using a cross-validation approach, as described in the methods:

"Cross validated median-split. To simulate an increase of signal-to-noise ratio, we removed sessions with low decodability. Importantly, to avoid circularity in our analysis we cross validated this selection in the following way. For each session, we split the trials in two halves. With the first half, we sorted the sessions by their decoding accuracy during early delay [0.2 - 0.4] s, and we selected high and low decoding sessions (median split). We then computed the average decoding accuracy along the trial for low and high-decoding sessions in the second half. We repeated this procedure 1000 times and then plotted the 90% C.I. of decoding accuracies for the high and low-decoding sessions."

With this selection of sessions, we are able to show robust decoding in the delay period for unattended, but not discarded memories (Fig. 3). This new result substantially strengthens our overall message and we want to acknowledge the reviewer for their suggestion. (see also response to Thomas Christophel and Vivien Chopurian, below)

The authors also point out that TMS reactivation may be qualitatively different, because simulation suggest that TMS *increases* across trial variability, and that this falls in line with the predictions of "a biophysical network model of memory reactivation from silent, synaptic traces". I thought this was

an interesting point, but it seems to sparsely described to really have a strong impact in the paper. Moreover, while being "potentially consistent" with that biophysical model is interesting, how strong is this evidence? I would be surprised if models that denied the role of "activity-silent" representations in working memory could not also be "consistent" with the finding that across trial variability is increased following TMS. But if the authors have a compelling argument that I should be surprised here, I think it should be clearly spelled out in the paper. If there is no strong argument, then I'd recommend tempering this particular conclusion. My view is that the really clear evidence in this study is the positive findings with alpha power in the pinging studies, and the simulations of how changes in SNR could explain the original findings in the pinging study. The other speculations regarding the potential causes of the TMS findings are not yet as convincing.

This insightful comment by the reviewer has led us to reframe our assessment of the plausible mechanisms underlying TMS-induced EEG reactivations, by also considering increases in decoding in attractor models that do not depend on 'activity-silent' mechanisms. We performed simulations in such a biophysical network model, where an ongoing neural representation is 'boosted' (i.e. increases tuning and therefore decodability) upon unspecific (e.g. 'pinging' or TMS) stimulation (Fig. S3). In such a network model, variability is reduced upon attractor-boosting (Fig. 4C, gray). The intuition is that the boosting is achieved through additional depolarization of all network neurons, which makes neurons fire more in the mean-driven than the fluctuation-driven regime, so that variability is reduced (Renart et al. Neural Comput 19:1, 2007, https://doi.org/10.1162/neco.2007.19.1.1). Instead, the increase in variability in the 'activity-silent' model stems from the fact that reactivations in this model emerge from the all-or-none triggering of a bump attractor, at the location primed by short-term plasticity encoding the silent memory. Such reactivations will occur randomly, either failing to trigger a memory, or triggering on slightly different neuronal populations each time, based on the fact that the short-term plasticity trace is a weak modulation over an otherwise noisy spiking network. We now added this intuition to the main text: "This is because reactivations in such attractor networks are an all-or-none phenomenon, and great variability is expected when triggering them from weak, decaying activity-silent traces in noisy spiking networks".

Following the reviewer's advice, we have now tempered our conclusions on TMS reactivations. We now conclude that TMS reactivations may depend on either activity-silent mechanisms (based on increased variability) or on attractor-boost mechanisms (assuming variability increases comes from TMS coil placement imprecisions).

Minor points:

1. The authors use the same analysis pipeline as the originally published papers, with the inclusion of alpha power instead of raw EEG amplitude. This is a powerful approach. However, the original analysis pipeline only included 17 posterior electrodes. It is possible that information about working memory could also be represented in other electrodes on the scalp. Therefore, decoding accuracy could actually be higher if the authors deviated from the original methods and included all electrodes in their models. It may be useful to include an additional analysis that addresses whether a model that includes all electrodes increases decoding accuracy of working memory representations.

We thank the reviewer for this constructive suggestion. Unfortunately we don't have access to the other electrodes (data shared in the original publication does not include them). However, we realized that our new analyses to improve low SNR and successfully decode from raw EEG (point

addressed above and in Figure 3) already addressed the reviewer's concern expressed in this point in particular.

2. In the current manuscript, the authors clearly show that alpha power tracks working memory representations in the time periods where the prior work suggested that there were no neurally active representations. This is the critical empirical pattern. That said, recent work suggests that alpha power and EEG voltage may play qualitatively different roles in working memory tasks:

Bae, G. Y., & Luck, S. J. (2018). Dissociable decoding of spatial attention and working memory from EEG oscillations and sustained potentials. Journal of Neuroscience, 38(2), 409-422.

Hakim, N., Adam, K. C., Gunseli, E., Awh, E., & Vogel, E. K. (2019). Dissecting the neural focus of attention reveals distinct processes for spatial attention and object-based storage in visual working memory. Psychological Science, 30(4), 526-540.

We thank the reviewer for noticing we were not discussing (or citing!) these important papers. We have now included a paragraph discussing the relationship of our results to these papers (in Discussion: "Finally, our results suggest that voltage and alpha power encode similar working memory content. ...")

So, it may be worthy of some discussion whether the active neural signal the present authors have identified might reflect a different aspect of working memory maintenance than do the patterns of activity in EEG voltage. This could be an interesting compromise between the original framing of the prior reactivation studies and the present framing.

We think that our new analyses showing a significant working memory code even in raw EEG (in contrast with the original publication) suggests that (at least in this case) voltage and alpha power may be reflecting similar stimulus codes, but voltages reflect a weaker code, possibly because of voltage baseline drifts in EEG. We have adapted our manuscript to reflect this view at various points. For instance:

(1) "In line with the hypothesis of an active but weaker representation ..."

(2) "Finally, our results suggest that voltage and alpha power encode similar working memory content"

3. The published data was baselined, and the authors reversed this baselining for their analyses. The authors should provide further explanation for why they reversed this baseline and should additionally discuss whether they were able to decode working memory representations with the published, baselined data.

Following the remarks by several reviewers, we have now included an extra figure (Sup Figure 3) to explain clearly what baseline we reverted. Essentially, our reasoning was that trial-by-trial baselining during periods in which stimulus may be represented (such as prior to the pinging stimulus, Fig. S3B bottom) complicates substantially the interpretations, as the mere baselining may be transfering code from one time point to another. This has been recently described in detail, with the general recommendation of using minimally preprocessed (filtering, detrending, baselining) EEG voltages for decoding analyses (Driel et al. J Neurosci Meth 352:109080, 2021).

4. The authors introduce fano-factors in Figure 3, but do not include a discussion of fano-factor anywhere else in the paper. Given the prominence of "△fano-factor" in their figure, the authors should include a brief description of fano-factors in the Method's section of their manuscript. This description will help bridge the literature that typically uses fano-factors to the literature that typically investigates alpha power activity in human EEG.

We thank the reviewer for pointing this out. We have now added a paragraph in the Methods section describing the fano-factor analysis.

Conclusions:

The authors make two main conclusions (1) working memory representations that were previously thought to be maintained in an activity silent state have been shown to be tracked via an active neural trace in the alpha band, and (2) visual "pinging" changes the ratio of signal-to-noise in EEG signals, thereby allowing active signals that may have been masked by noise to be detected. This work provides an important new interpretation of a highly influential set of studies, and I believe it would have a strong positive impact in the literature that will generate vigorous follow-up work. Their conclusions, however, would be strengthened by addressing the above comments.

We thank the reviewer for their very useful comments and alternative points of view, in particular the one that motivated our attractor-boost model. To reflect this, we have included “Reviewer #2” in the Acknowledgments section.

Reviewer #3, Thomas Christophel and Vivien Chopurian: The authors report a short reanalysis of EEG data from recent studies investigating reactivation (via 'pinging') of supposedly 'activity-silent' mnemonic traces during working memory. This reanalysis shows that alpha band activity carries an active trace of memorized content, in contrast to this prior work. Additional reanalyses concern the nature of the 'pinging' effects reported in this and other prior work. The manuscript argues for differential effects of 'pinging' using either sensory mask-like stimuli and TMS on the reduction and increase of across-trial EEG variability, respectively.

The finding that data in a core study seemingly supporting 'silent' working memory contains evidence for active neural representations alone is critical to the field and beyond. This finding is robust and consistent with previous work and without any doubt deserves the attention given here. There are opportunities to elaborate more on this finding and its relationship to other work, but it is convincing as is. The additional reanalyses and their interpretations highlight some explanations for 'pinging' effects, but several concerns let me doubt the conclusions drawn here.

We will outline our suggestions, concerns, and recommendations in the following sections. Beforehand, we would like to emphasize that our expertise primarily lies in related fMRI work.

Major:

* Our concerns solely relate to the second part of the manuscript which argues that "visual pinging reveals an underlying active code by quenching EEG noise". As the authors outline this is indeed a plausible explanation, but it is questionable whether the data analyses reported are sufficient to support the hypothesis in a substantive way.

As the authors outline, a reduction in trial-by-trial variability is the expected outcome of introducing a stimulus into a system that is constant across trials. Simply put, stimulation (like a 'ping') can be seen to replace endogenous by with exogenous activity. If this exogenous activity is constant across trials, variability across trials is reduced. It is equally plausible that in a decoding analysis, the response related to a memorized item is more easily decoded when noise is reduced. The 'noise' in this decoding analysis and the 'trial-by-trial variability' in the analysis provided, however, are not the same. On the contrary, the trial-by-trial variability is composed of both the noise and the signal used for the decoding analysis. This becomes obvious as one considers the fact that subjects memorize different orientations on different trials, making the signal itself vary across trials. How different components (ping signal, mnemonic signal, and residual noise) are aggregated to form the overall EEG signal is unknown, but here we see little indication that trial-by-trial variability can be used as an exclusive indicator of the residual noise component. For these reasons, we do not see how the trial-by-trial variability analyses provide evidence in favor or against the 'quenching noise' hypothesis.

We thank the reviewers (Thomas and Vivien) for pointing out this unclear point. The reviewers are right that our variability calculations do not factor out the effects of stimulus variation. Perfect factoring out is problematic because there are two stimuli presented simultaneously, each with a different random (continuous) orientation. Thus, as the reviewers argue, the absolute value of voltage variance across trials contains variance both from presented stimuli and from the noise. For this reason, we designed our analyses so as to consider only changes in variance taking the variance in the period immediately preceding the pinging stimulus as a reference. Before the appearance of the pinging stimulus, the variance in presented stimuli is the same as during the pinging, so this difference in variance is capturing explicitly only the additional variance induced by the pinging stimuli. Because there is no variation of pinging stimuli across trials, our measure is capturing changes in residual noise variance across trials. Thus, our analysis addresses the concerns raised by the reviewer. However, we acknowledge that the earlier form of the manuscript was not clear about this reasoning. We have explained this briefly in the Methods section of the revised manuscript:

> “Across-trial variability analyses. We computed variability (var) across trials of the raw voltage traces (trials x sensors x time). Before averaging across sensors, we detrended the data using the function detrend to account for any drift in the signal. Finally, we computed the percentage of variability change (var) relative to the baseline period of 2 s before the pinging stimulus (b): Δvar =(var - b)/b. This referencing ensures that the increase in variability can be attributed solely to the pinging and not to other factors that are common to both pre and after pinging, suchs as stimulus variabitlity.”

* This problem becomes even clearer when considering the trial-by-trial variability in Rose et al: In Rose, not only does the content vary across trials, but the ping is variable across trials, too (in the EEG Exp 2). Considering that this means that on different trials the TMS coil is pointed at different parts of the brain, one might expect to see it in the absence of any mnemonic or cognitive activity (e.g. in a lifeless neuronal substrate).

We see the point of the reviewers, and indeed this is an important element of TMS studies that must be considered. However, we want to stress that in the EEG Exp 2 by Rose et al. (2016) TMS targeted different brain areas **in different blocks of the task**. Since our variance calculations are done separately for different task blocks, and then averaged, the variance corresponding to targeting different areas (which would indeed be massive) does not appear in our measurement. However, holding a TMS coil at a fixed position relative to the participant’s brain (even with a

neuronavigation system) is challenging and we should count with trial-by-trial localization imprecisions of the exact TMS stimulation point, and this could induce also some variability in EEG voltages that we would capture in our analysis. Based on this caveat when interpreting the TMS result, we now declare that we do not know what mechanism underlies TMS-induced EEG reactivations, but our analyses suggest that it could be an activity-silent mechanism.

At the end of Results:

> "Such increase in across-trial variability is in accordance with the activity-silent working memory model presented before (Fig. 4C, black), thus supporting the interpretation of TMS EEG reactivations as signals recovered from activity-silent traces [5,6]. However, a note of caution is in order: the difficulty in precisely locating the TMS coil in different trials may strongly contribute to increased EEG variability, which could mask the true effect of TMS on EEG variability."

1st paragraph of Discussion:

> "Based on the difference in behavioral impact of these two perturbation protocols (visual pinging does not affect working memory behavior, but TMS does), we speculate that visual pinging may increase EEG decodability via reduced across-trial variability or by transient boosting of active attractors, while TMS-induced reactivations would be supported by activity-silent mechanisms. "

Notably, this also provides a relatively straight forward explanation for the 'reactivation' in Rose et al. (in their Exp 2, at least): The TMS itself already carries information about the memorized content, which makes decoding the content after the pulse a trivial finding.

If we understand correctly the analysis in Rose et al. (2016), decoding the presence or absence of a given stimulus category was performed in individual sessions, when TMS location was held fixed. Within each session, the presence or absence of each category was also balanced across trials, so there was no obvious bias related to the specific area being targeted with TMS. Variance in TMS location would therefore not be a possible explanation for the increased decoding after the TMS pulse.

* What It is essential here, is that a critical finding (active representations in the Wolff data) is not hampered in impact, by jumping to conclusions in the second part of the manuscript. While the 'quenching noise' interpretation is a plausible one, alternative interpretations need to be considered. One simple interpretation is that 'pings' increase overall alertness or modality-specific attention which in turn increases the signal of any memorized content. This could be seen as a mechanism to counter interference by distracting stimulation (see Bettencourt and Xu as well as Rademaker et al., in particular, the difference between expected and unexpected distraction in Bettencourt and Xu).

We thank the reviewers for this point. We now provide additional evidence in favor of a role of quenching noise in the increase of EEG decodability, in particular providing new data from EEG, but we also elaborate the alternative hypothesis that the reviewer presents, backed up by explicit simulations:

*EEG link of across-trial variability with reactivations*

We selected trials based on how accurately we could predict the memorized item's orientation in the pinging period, making two classes: high decoding and low decoding trials. Then we computed the

variance change for trials in each of these two classes. The prediction of the quenching noise model is that high decoding trials will be characterized by lower voltage variance than low decoding trials, specifically during the pinging period. This is precisely what is observed in the data of Wolff et al. (2015) (new panel in Fig. 4, Fig. 4B). For the other experiment (Wolff et al. 2017) we did not see significant differences, but in this experiment reactivations are not really apparent in the debaselined data, so trial-by-trial decoding to classify low and high decoding trials may be too noisy for this analysis (Fig. S3). This lends support to the ‘quenching noise’ interpretation.

*Attractor-boost model of reactivations*

We also provide now another possible explanation for increased decodability following pinging, inspired by the reviewers’ suggestion. We simulated a network model (“attractor-boost model”) without short-term plasticity (thus factual reactivations are not possible) (Fig. S2) and we found that for this model, the prediction in terms of variability is also a reduction (Fig. 4C, gray) upon pinging-induced increase in tuning (Fig. S2). This model thus also appears to be a possible interpretation of the visual pinging effect, at the neural level and we do explain both possibilities in detail.

In Results:

> “Alternatively, there is another interpretation of pinging-induced increases in EEG decodability consistent with our findings. (...) Also this mechanism would be consistent with these data, as it shows reduced variability (Fig. 4C, gray), concomitant with boosted attractor tuning (S2 Fig.).”

In Discussion:

> “Based on this substrate for working memory, visual pinging may increase EEG decodability through (1) ping-induced reduction in across-trial variability, and/or (2) ping-induced boosting of attractor tuning. We further compare visual pinging with TMS perturbations, and we find qualitative differences suggesting different underlying mechanisms. Based on the difference in behavioral impact of these two perturbation protocols (visual pinging does not affect working memory behavior, but TMS does), we speculate that visual pinging may increase EEG decodability via reduced across-trial variability or by transient boosting of active attractors, while TMS-induced reactivations would be supported by activity-silent mechanisms.”

As for the simulations of the attractor-boost model, we have opted to put them in the supplementary material because similar models have been put forward in previous publications (Edin et al. 2009; Schneegans and Bays 2017)

This highlights that a robust explanation of 'pinging' effects must consider both enhancing ('pinging') and disrupting ('distracting') effects of stimulus presentations during working memory delays which are likely to jointly affect neural decoding in different ways across studies.

This is a very interesting point, which unfortunately escapes the scope of the current manuscript. We agree that an understanding of how intervening stimuli affect working memory is a major question in the field and deserves coordinated efforts. In this manuscript we are focusing only on the previously reported effect of visual pinging and even if not addressing the broader picture, including distractor effects for instance, we do think that providing possible specific neural mechanisms is a valuable contribution to generate mechanistic hypotheses for future experiments.

Minor:

* The main finding of the paper is a rather simple but important one: If you run the same analysis as Wolff et al. (2017) with the same data but using Alpha power rather than raw data, then you can decode unattended items, which were not decodable before. One potential avenue to make this manuscript even more insightful would be to provide further analyses that help explaining this finding.

As the authors mention, raw EEG power already carries information about a memorized item in Wolff et al. (2015) when combining 'pinging' and 'no-pinging' trials prior to the 'ping'. The same analyses split half for 'pinging' trials results in an occasional null result ('long trials only'). This highlights the trivial but apparently forgotten idea that statistical power might be the essential determinant of whether one is likely to find a positive result in a given study. The change from raw signal to Alpha power might simply constitute an increase in per trial effect size (like other forms of filtering and smoothing). In other words, the question is whether the difference between raw and Alpha -based analyses represents a qualitative difference in the underlying signals (between UMI and AMI) or simply a quantitative difference in the strength of representation.

We suggest quantifying this effect by running raw-EEG and Alpha decoding for both studies and 'simulating' studies with different sample sizes (by randomly removing subjects from a given study) or even different number of trials (e.g. split half). We also suggest running analyses combining decoding accuracy across time-points (e.g. averaging across the delay) on a given trial and/or combining trials prior to the decoding analyses (similar to run-wise beta decoding in fMRI) to increase statistical power for raw analyses. We want to stress that these are suggestions to better explain an already relevant findings rather than analyses necessary to support the finding. Not all these suggestions might be feasible for the datasets available.

We have included two new main figures (Figure 2,3) and changed the text to include most of the reviewer suggestions (focusing on Wolff 2017, rather than 2015). Based on these new analyses we do find that there is decoding of the unattended item in the delay in raw voltages, thus supporting the view proposed by the reviewers that, rather than an alpha/voltage distinction, we are looking here at a problem of statistical power, which affects especially voltage traces. Based on these new insights, we have now changed the framing of our manuscript to emphasize this and to remove the focus from alpha power per se. In our view, this has strengthened our message and we want to sincerely thank the reviewers for these excellent suggestions.

In Results:

> "In line with the hypothesis of an active but weaker representation of the unattended items, recent studies (Christophel et al. 2018; Iamshchinina et al. 2021) show that lack of decodability for unattended working memories can be overcome by increasing statistical power (e.g. sample size). We wondered if…" and rest of the paragraph

* One final recommendation is to investigate the differential involvement of different electrode positions in the decoding of AMIs and UMIs. We want to be very clear, however, that is more driven by the reviewers' curiosity than anything else.

As per the reviewers' suggestion (see also response to Reviewer #1), we have looked into the format of unattended (UMI) and attended (AMI) memories. To investigate this, we computed "tuning curves" by averaging alpha power across stimuli (analyses 1). In an alternative analysis (analyses

2), we also computed the std across stimuli for each electrode as a rough measure of code strength for each electrode. We then correlated both these vectors of PMI vs UMI for each subject. For both analyses, we find a positive correlation between the representations (r>0.3) but only one session stronger than correlating codes computed with shuffled stimuli (p=0.01, p=0.03 t-test against shuffle). This means that electrodes tend to use the same code (for session 1 and inconclusive for session 2) for AMI and UMI (analyses 1) and that similar electrodes encode AMI and UMI (analyses 2). Because this is not the focus of our paper we opted to not include this analysis as it would imply discussing a topic that was not addressed in the current manuscript.

* "[…] moving electrodes using the python function signal.detrend on each subject variability." We assume this refers to the function in the scipy package and the linear detrending option thereof, please specify. There also might be a typo here ("each subject's").

Corrected, thank you for noticing it.

* "Mahalanobis distance between all possible pairwise combinations of the orientations and thus form a tuning curve." Please specify how these pairwise distances are turned into a tuning curve. An array of pairwise distances is typically referred to as a representational dissimilarity matrix.

Since we adapted the original code, we wanted to have a similar methods description. However, we agree with the reviewers that “tuning curve” might be a misleading name for what is more resembling a dissimilarity function and we have changed the methods accordingly.

* "We realized that an earlier study had reported that attended spatial memories were decoded more reliably from EEG alpha power than from raw voltages [16]." This might be related to our primary expertise in fMRI data, but when reading Foster et al. (2016), our understanding is that they differentiate between evoked and total power not raw voltages and Alpha power. See David et al. (2006): "In short, evoked responses can be characterized as the power of the average; while induced responses are the average power that cannot be explained by the power of the average." 'Total' power would then be both components combined which (in our understanding) is what is used, here. Naturally, there is a relationship between evoked power and evoked responses in preprocessed (rather than raw) EEG data, so the results by Foster et al. still can serve as a motivation for the reanalysis in the current manuscript. The authors should however clarify what prior work found.

We thank the reviewers for this very pertinent point. Indeed Foster et al. (2016) did not compare alpha and raw voltages but total and evoked alpha. So the point of that paper is that alpha amplitude carries location information in the delay, but the phase of alpha oscillations is not locked to task events so averaging on the time domain reduces decodability strongly. This is the point that motivated our analyses, and we have now clarified it in the text:

> “We realized that an earlier study had reported that (attended) spatial memories were decoded more reliably from EEG total alpha power than from evoked activity [19].”

* "Despite their relevance for upcoming memory-guided behavior, currently unattended memories cannot be robustly decoded from raw EEG voltage traces [6,7] (Figure 1a, red)." This might be somewhat misleading as prior work only showed that they were not able to decode from

raw voltages which might simply be a false negative. The prior work does not show that UMIs "cannot" - in principle - be decoded.

We agree with the reviewers entirely. In fact, our manuscript is about calling for caution when making such claims. We thank the reviewer for noting that we too were making a similar logical mistake. We have changed the word **"cannot"** to **"could not"** (here and in the rest of the paper), or explicitly stated that **we fail** to detect decoding

* "Furthermore, it challenges the current view on the role of alpha power during working memory maintenance [18], which would suppress immediately irrelevant memories [19]." Here, it might be worth noting that suppression might lead to inverted tuning which could be decodable equally well than non-inverted tuning. This means that being able to decode an item does not necessarily mean that it isn't suppressed or that the signal one decodes isn't suppressive in nature. (See the different works by Lorenc and Postle and van Loon). Notably, there is plenty of debate about these inverted tuned representations, so it is unclear to me whether to include the debate here.

We fully agree with the reviewer (and Reviewer #1) that this sentence was opening many interesting side-debates. In the previous manuscript, the emphasis on alpha may have justified some extended discussion on the role of alpha in working memory. However, in the current revised version, the fact that we can now observe decoding both in alpha and raw voltages reduces notably the interest of such debates on alpha for this manuscript. We therefore opted to remove this sentence from the manuscript altogether.

* "Despite apparent similarities, TMS reactivations impact working memory performance [5,6], while pinging does not [7]." Here it is worth noting that (by our count) in Rose et al. only one out of three TMS experiments show a behavioral effect of the TMS (one out of six possible effects in exp. 4 at p = 0.01, which is not reanalyzed in the current manuscript). Overall effects of interfering stimulation with distractor or TMS are rather scarce, so we caution to interpret this apparent difference more carefully.

We want to note that the effect of TMS on behavior in working memory tasks goes beyond Rose et al (2016) (for example Zokaei et al. 2014; Machado et al. 2021; Fulvio and Postle, 2020; Barbosa et al, 2020). We are thus confident that TMS does have an impact in performance in working memory tasks (especially for serial dependence, for which we ran a replication, pre-registered study (Barbosa et al., 2020), which in addition is consistent with reactivation models).
To further support the contrast between TMS and pinging in terms of behavioral impact, we have focused on serial dependence for the purpose of this rebuttal, based on our conviction that for this specific effect TMS perturbations in the fixation period increase serial dependence (Barbosa et al 2020). We have not been able to test the effect of pinging on serial dependence in the datasets of Wolff et al. (2015, 2017) because pinging was present on every trial, so we ran an online experiment where we omitted pinging on some trials randomly. We collected data for n=112 participants and we found that there was no effect on serial dependence ($p>0.5$, three-way interaction), in contrast to the robust TMS effect found in n=20 participants with a replication study (Barbosa et al. 2020). Thus, we conclude that the point that visual pinging has little behavioral impact in conditions where TMS has robust effects is upheld by these analyses, and we propose to maintain our reasoning in the revised manuscript.

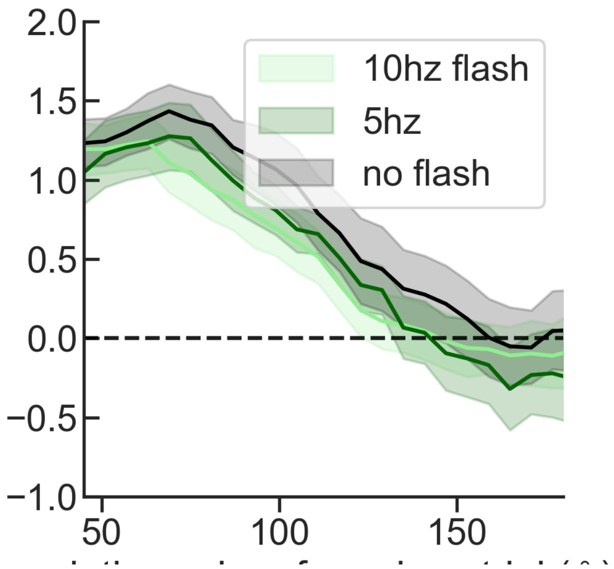


Serial biases computed for subjects (n=112) with at least 100 correct trials (error < 45º). In contrast with the TMS experiments, there was no apparent effect of the non-specific stimulus on serial biases (p>0.5, three-way interaction)

* "Decoding from raw voltage or alpha power was done with the exact same code, but preprocessing the data differently.": The second sentence seems to miss a "with" or we suggest changing the voice from passive to active

Corrected, thank you for the suggestion.

* "Each 'trial' trace was simulated as a slope $\alpha$, different for each stimulus ($\alpha1 = 1$ and $\alpha1 = 1/2$) on top of noise sampled from a normal distribution $\xi$, with mean 0 and standard deviation 1." '$\alpha1 = ½$' should probably be '$\alpha2 = ½$'. It would be helpful to explain the selection of these model parameters (why 1 and 1/2)? Further it might be helpful to spell out the simulation a little (e.g. What are the two items, what is the data being simulated, where does the data start, where is 0 ….), it took us more time than necessary to get what the authors are doing here and why. Arguably, however, the whole simulation can be seen as demonstrating the trivial fact that SNR means Signal to Noise ratio, but we'll leave it to the authors judgement whether explaining this is necessary.

Typo corrected, thank you for noticing it. We also agree that this is a rather complicated description and figure for such a simple message, so we have removed this figure. Instead, we make this point with EEG simulations, together with our illustration of the baselining (Fig S3).

We wish you all the best for this fascinating project.

Thomas Christophel and Vivien Chopurian

Thank you very much for the extremely thoughtful and constructive suggestions. We genuinely believe that the manuscript message has become stronger and clearer and that was in large part by following your suggestions. We have included your names in the Acknowledgments section. Please let us know if you would rather not be named there.
