# Supplementary material for: Pinging the brain with visual impulses reveals electrically active, not activity-silent, working memories
Source: PLoS Biol. 2021 Oct 21;19(10):e3001436. doi: 10.1371/journal.pbio.3001436 (PMC8641864; doi:10.1371/journal.pbio.3001436)
Supplement: Attachment — Submitted filename: Barbosa_et_al_rebuttal.pdf [file pbio.3001436.s005.pdf]

Point-to-point reply

Reviewer #3, Thomas Christophel & Vivien Chopurian: The authors provide an extensive response to our comments. The revised manuscript includes several additional analyses and simulation which critically add to the prior work. The updated main finding shows that unattended memory items can be decoded from both raw and frequency transformed EEG data. Moreover, 'reactivations' by 'pinging' appear to depend on the presence of an active raw EEG trace of the unattended item. Finally, as an alternative explanation for pinging effects, they introduce an attractor-boost model which appear to equally explain their results. The authors have broadened their interpretation of their initial findings in the light of these additional findings and discuss possible underlying mechanisms.

These changes greatly improve the quality of an already noteworthy study. Some open points remain. These concerns predominantly relate to the interpretation of Rose et al., which play a lesser role in the revised manuscript. We will outline our suggestions, concerns, and recommendations in the following sections.

Minor:

1. Data from Rose et al. (2016) is reanalyzed for the current study and plays a part in the conclusions drawn by the authors (e.g. in the discussion: 'We further compare visual pinging with TMS perturbations, and we find qualitative differences suggesting different underlying mechanisms'). The authors however did not analyze the TMS-EEG data in the same way as the Pinging data and conclude different mechanisms based on one analysis looking at difference in variation measures between the two sets of experiments.

The authors now acknowledge that variation in the TMS site might result in an increase in variability but appear to contend that reactivations (meaning an interaction of the TMS with the respective conditions) are the more probable cause of this increase, as the TMS itself should average out due to the block-wise nature of their analysis. When looking at the data directly, however, we see that the by far largest increase in variability (so large that it evades the y-axis on the respective plot) occurs at 0ms relative to the pulse. In other words, the largest change in the EEG measurements is the pulse itself which - according to the authors - should have been averaged out. Hence, we would attribute the differences in variability found between pinging and TMS to variations in the TMS itself not a difference in the underlying mechanism of the reactivation which might be sensibly explained by boosted active representations or even by noise-quenching (which would be overshadowed TMS-related noise). We suggest a more careful interpretation of these differences in variability and possible underlying mechanisms.

We agree that these are relevant concerns. We ignored this peak of variability because it is most likely due to variance in TMS-induced artifacts on EEG recording, due to saturation of EEG amplifiers during the TMS pulse. This interference, not related to brain responses, is

known to vary strongly with coil orientation (Sekiguchi et al. 2011 https://doi.org/10.1016/j.clinph.2010.09.004). So, although we now display the full curve in the figure for transparency, we do not think that short-latency effects of the pulse can be interpreted in this study. (As a side note, we noticed that six out of 54 sessions had outlier TMS artifacts with extremely high increase of variance and we removed those sessions from the analysis to avoid biasing our analyses). In light of the concerns raised by the reviewers - in particular how TMS-locked increase in variability might be long lasting and thus mask eventual variability dynamics induced by reactivations we have also extended our previous note of caution (end of Results):

“However, a note of caution is in order: the difficulty in precisely locating the TMS coil in different trials may contribute to increased EEG variability by virtue of the long lasting effect of the TMS pulse on neural excitability [48]. This could mask EEG signals reflecting TMS-induced reactivations.“

2. For the across-trial variability analyses, please clarify the block-wise nature of this analysis in the methods section.

Done, thank you for the suggestion.

3. Please extend the y-axis for the variability analyses such that the reader can see the extend of the peak for the data from Rose et al. (2016).

Done, thank you for the suggestion.

4. "We then tested the influence of sample size on decoding estimates in each condition, by varying the number of sessions and trials included in the analyses. This showed a striking difference between unattended and discarded memories: while increasing the sample size in the unattended condition resulted in a monotonic increase of t-value, it did not for discarded memories (see diagonal in Fig. 2). This result suggests that increasing the number of sessions would lead to decodability of unattended memories, but not of discarded memories." This monotonic increase might be less striking than one might think. In a bootstrapping procedure like this, the closer a randomly drawn sample of trials gets to the full set the closer it will approximate the results with all trials included. For unattended items this results appears to be $p < 0.1$ whereas for discarded it is $p \sim 0.48$, which appears to be the main difference here.

The reviewers are right as this differential effect is somewhat expected, so we have toned down our results presentation. We now present Fig. 2 as a visualization of how our analyses depend on sample size, as done in other similar situations (Hajonides et al. Neuroimage 2021) and as per the suggestion of these reviewers.

Notably, $p < 0.1$ would constitute a significant result if tested using a one-sided test ($p < 0.05$) and we see little reason to perform a two-sided test (because below chance classification is

neither hypothesized nor plausible unless confounds affect the cross-validation, see Görgen et al., 2018, Neuroimage).

We are sorry for the misunderstanding. In Fig 2 and Fig 3 we are indeed doing one-sided but this was not properly indicated for Fig 2. We have now updated text to be fully coherent. Thank you for noticing it.

Please provide a detailed explanation of this analysis in the methods section. Furthermore, it is unclear to me what time-points are used for these plots (i.e., what $p < 0.1$ refers to).

Thanks for noticing this omission, we have now added this info in the Figure caption.

5. Throughout the manuscript the work by Panichello and Buschman (2021) is cited as evidence for active representations of unattended items found in intracranial recordings. In our view, this study cannot be easily interpreted as such evidence as (1) these items can be discarded after the cue and (2) these signals were only observed until 500 ms after the cue when they were in decline (i.e., there might have been too little time for them to reach baseline), At least in our reading, Panichello and Buschman might not agree with such a characterization of their results.

The reviewers are right that this work does not show representations of unattended working memories, but of discarded, irrelevant memories. We have changed this in our text, to be more precise and we thank the reviewers for this observation. On the other hand, the evidence of this study does show that the monkeys kept these irrelevant memories in robust active representations until the response, with little indication of decay in most cortical areas.
